# Determinants of the acceptability of health problems in different ages: exploring a new application of the EQ VAS

**DOI:** 10.1007/s10198-019-01060-3

**Published:** 2019-05-20

**Authors:** Zsombor Zrubka, Zoltán Hermann, László Gulácsi, Valentin Brodszky, Fanni Rencz, Márta Péntek

**Affiliations:** 1 Department of Health Economics, Corvinus University of Budapest, Fővám tér 8, 1093 Budapest, Hungary; 2 Doctoral School of Management, Corvinus University of Budapest, Fővám tér 8, 1093 Budapest, Hungary; 3 Centre for Economic and Regional Studies, Hungarian Academy of Sciences, Tóth Kálmán u. 4, 1097 Budapest, Hungary; 4 Centre for Labour Economics, Corvinus University of Budapest, Fővám tér 8, 1093 Budapest, Hungary; 5 Premium Postdoctoral Research Program, Hungarian Academy of Sciences, Nádor u. 7, 1051 Budapest, Hungary

**Keywords:** Acceptable health states, EQ-5D-3L, EQ VAS, Priority setting, I10

## Abstract

**Background:**

We aimed to determine the acceptability of non-perfect health states with age using the EQ VAS and analyse the influencing factors.

**Methods:**

We conducted a cross-sectional survey on a convenience sample from the general population (*N* = 200). Respondents were asked to indicate on the EQ VAS the health states that are still acceptable for ages between 30 and 80 years in 10-year intervals (VAS acceptable health curve, AHC_vas_). We recorded respondents’ current health, health-related lifestyle, demographic background and explored the reference person they imagined when evaluating acceptable health states. We evaluated the AHC_vas_ by estimating linear multilevel models including a random intercept (estimated at age 30) and a random slope for age.

**Results:**

AHC_vas_ scores were available for 194 respondents (mean age = 42.8 years, range 19–93, 58% female). For ages of 30, 40, 50, 60, 70 and 80 years, mean AHC_vas_ scores were 93, 87, 80, 73, 65 and 57, respectively. The decline of AHC_vas_ was linear with age. Respondents’ age, health status, lifestyle and health-related experiences, as well as their reference point taken (e.g. imagining themselves, others or both during the valuation task) influenced significantly the acceptability of health problems.

**Conclusions:**

When measured with the EQ VAS, health problems were increasingly acceptable with age. Capturing well the individual variability in the assessment of acceptable health states at different ages, the EQ VAS is a useful addition to EQ-5D-3L descriptive system-based measures of acceptable health.

**Electronic supplementary material:**

The online version of this article (10.1007/s10198-019-01060-3) contains supplementary material, which is available to authorized users.

## Introduction

Fiscal sustainability of healthcare has become a key challenge of developed economies, resulting in an increasing focus on the efficiency of public spending on healthcare [1]. To tackle this challenge, among several policy options, decision-makers may need to be increasingly selective when defining the benefit basket covered by public reimbursement systems [1].

Can this challenge be met by considering the preferences of the general population as a beacon for decision-making?

Standard economic analysis uses perfect health as a reference point for health gains and applies the same weights to QALYs across the full range of disease severity or age of the target population. However, when making decisions about the public funding of various treatment options in scarcity of healthcare resources, alternative approaches may be considered, which differentiate QALY gains on the basis of various theories of distributional justice [2]. The ‘fair innings’ principle argues on the grounds of egalitarian ideology about placing greater weights on QALYs in younger individuals, who have not yet had a fair share of lifespan as compared to older individuals [3]. The ‘worse off’ principle on the grounds of prioritarian ideology favours QALY gains in more severe disease states over less severe ones [4], while other principles may favour the maximisation of lifetime QALYs or interventions with the greatest benefit [5]. Using sufficientarian reasoning, Wouters et al. recently explored the principle of differentiating QALY gains based on the acceptability of health states. Instead of being concerned with inequalities, sufficientarianists propose that it is morally important for everyone to have just enough [6]. It has been shown in empirical studies on the Dutch general population [7, 8] and a sample of Hungarian patients with rheumatoid arthritis (RA) [9] that people have internal reference points, against which they compare the acceptability of certain health states via a simple acceptable—not acceptable judgement. The reference points depend on age, suggesting that more health problems are acceptable in older ages [7–9]. According to sufficientarian reasoning, it is desirable to live above the acceptability threshold. Therefore, highest utility could be attached to health gains, that move patients from unacceptable to acceptable health states (AHSs), while depending on the application other sufficientarian criteria, zero or lower utility level could be attached to those health gains below or above the acceptability threshold, that do not cross the reference line [2].

The relative simplicity of the cognitive evaluation task makes the measurement of AHSs a compelling approach. The EQ-5D-3L descriptive system [10] provides a standardised framework for measuring AHSs, which is an important feature for public decision-making [11]. However, rendering a binary acceptable/not acceptable status to all 243 discrete health states across several age-groups poses a feasibility challenge for the comprehensive evaluation of AHSs. To overcome these challenges, previous studies investigated AHSs in different ages separately by each dimension of the EQ-5D-3L [7–9]. These studies left uncertainty about the acceptability of simultaneous health problems in more than one dimension. The acceptability of combined health problems was jointly evaluated using only a few health profiles by Wouters et al. [7]. Although acceptable health may serve as a reference point for priority setting, it requires further methodological exploration. Wouters et al. pointed out that despite the appealing concept, the challenge of finding a morally acceptable and practically feasible acceptability threshold hampers its application in real practice [2, 7].

Building on the results and unanswered questions of previous empirical research using the descriptive system of EQ-5D-3L instrument [7, 8], we explored AHSs using the EQ VAS. The EQ VAS is a feasible and reliable instrument for the evaluation of health states [12]. We assumed that although the EQ VAS does not inform about the details of subjective criteria when evaluating the acceptability of health states, it may provide meaningful point estimates about where the internal reference health states fall relative to the extremes of best and worst imaginable health. Furthermore, the EQ VAS provides a single score about the individuals’ global evaluation of health, not affected by the properties of the EQ-5D-3L descriptive system and index values, which reflect the average societal preferences attached to discrete health profiles [12].

Our primary goal was to explore AHSs using the EQ VAS instrument and to analyse the differences compared to the assessment based on the descriptive system of the EQ-5D-3L. Moreover, we aimed to explore how AHSs are influenced by the health status and socio-demographic characteristics of respondents. Another novelty of our study is that we investigated how the reference person imagined by respondents during the evaluation exercise affected the acceptability of health states.

## Methods

### Study sample and design

Between January and March 2018, we conducted a cross-sectional survey on a convenience sample of 200 subjects from the Hungarian general population. We obtained the approval of the Medical Research Council of Hungary (ID: 5111-2/2018/EKU). Respondents were informed, and provided written consent. Data were collected anonymously. The acceptability of health problems was assessed via computer-aided personal interviews separately by EQ-5D-3L dimensions as well as by joint evaluation of EQ-5D-3L profiles using an adaptive testing algorithm. We recorded socio-demographic and health-related data using a paper-and-pencil questionnaire including the EQ-5D-3L, as well as the evaluation of AHSs by an adapted version of EQ VAS. The electronic and paper questionnaires were joined by a common code, retaining the anonymity of respondents.

### Socio-demographic and health-related data

Respondents’ age and gender were recorded, and three main age groups (18–43, 35–64 and 65+ years old) were formed for the analysis. The three education categories (low: primary, middle: secondary, high: tertiary) were based on the highest completed level of education. We recorded the lifespan of close relatives as well as respondents’ informal caregiver experience. We assigned informal caregiver status to individuals who provided at least 6 weeks of informal care over the past 10 years. We also asked health-related lifestyle questions: weight and height for body mass index (BMI), smoking, alcohol intake and physical activity. We considered the following lifestyle parameters as risky: BMI ≥ 25 [13], smoking at any quantity [14], ≥ 7 drinks per week or ≥ 3 drinks per any single day for women and ≥ 14 drinks per week or ≥ 4 drinks per any single day for men [15], and moderate physical activity < 150 min/week [16].

### The EQ-5D-3L instrument

We recorded respondents’ current health status using the paper-based validated Hungarian version of the EQ-5D-3L instrument [10]. EQ-5D-3L is a generic quality-of-life instrument, which consists of two parts [10]. The descriptive system assesses self-reported health in five dimensions: mobility, self-care, usual activities, pain/discomfort and anxiety/depression. Respondents are asked to describe their current health in each dimension with one of the following three categories: no problems, some problems and severe problems. The descriptive system defines 243 (3^5^) distinct health states, denoted by a five-digit profile comprised of the problem levels in each dimension. (e.g. 21,113 indicates moderate problems with mobility and severe problems with anxiety/depression with no problems in other dimensions.) The EQ-5D-3L index scores (utility values) attached to each health state reflect the preferences of the general population. The EQ-5D-3L index score of 1 represents perfect health, 0 represents death, and negative values represent “worse than dead” health states [17]. Due to the lack of a Hungarian EQ-5D-3L value set, we used the time trade-off (TTO)-based value set from the UK, which is the most frequently applied EQ-5D-3L value set in the Central and Eastern European (CEE) region [17, 18].

The second part of the instrument is a 20-cm visual analogue scale (EQ VAS) ranging from 0 (worst imaginable health) to 100 (best imaginable health). While the EQ-5D-3L descriptive system measures core dimensions of health-related quality of life to provide a single index that reflects preferences of the general population, the EQ VAS reflects the self-rating of the respondents’ overall health including aspects without limiting to the five EQ-5D-3L dimensions [19]. We assigned respondents with one standard deviation (SD) below the mean EQ VAS of the sample to the “poor health” group, respondents within ± 1 SD around the mean to the “average health” group, and respondents with one SD above the sample mean to the “good health” group.

### Measuring acceptable health states

We performed computer-assisted personal interviews to assess the acceptability of health states in different ages between 30 and 80 years in 10-year intervals. To allow a clear separation of these ages from respondents’ own age, we will use “age_AHS_” notation when referring to the hypothetical ages used to assess AHSs. In addition to separate evaluation of AHSs by the dimensions of EQ-5D-3L, we applied two novel evaluation methods: (1) joint evaluation of the acceptability of EQ-5D-3L health profiles using an adaptive testing algorithm, and (2) assessing AHSs by the EQ VAS. Reporting the results of the adaptive testing and joint evaluation is beyond the scope of this paper.

We assessed AHSs via separate evaluation of problems by dimensions of the EQ-5D-3L descriptive system according to the methods applied in previous studies on the Dutch general population [7, 8] as well as in Hungarian patients with rheumatoid arthritis [9]. In short, respondents were asked to indicate beyond what age they consider different levels of problems acceptable in each dimension of the EQ-5D-3L. The sample question for the mobility dimension is depicted in the Online Resource (Supplementary Fig. S1). For each respondent, we constructed acceptable health curves (AHCs) by the methods described by Wouters et al. [7]. First, we assumed that all health problems, that were considered acceptable in a certain age_AHS_ separately, would also be acceptable in combination. Therefore, we aggregated the individual responses on each EQ-5D-3L domain into a single EQ-5D-3L health profile for each age_AHS_, and attached the EQ-5D-3L index value for these aggregated health profiles. We will refer to this method using the term “aggregate acceptable health curve (AHC_aggregate_)” [7–9]. Alternatively, we assumed that respondents would only consider health problems acceptable in each domain with perfect health in mind for the remaining four domains. Therefore, for each age_AHS_, we also constructed AHCs using the lowest EQ-5D-3L index value among the possible EQ-5D-3L profiles having acceptable problems in a single dimension. We will refer to this method using the term “worst acceptable health curve (AHC_worst_)”. For example, if a respondent indicated moderate mobility problems and severe anxiety/depression acceptable from age_AHS_ 60 during separate evaluation, then the 0.345 EQ-5D-3L index of the aggregate profile 21,113 would be used in AHC_aggregate_; while from the profiles 21,111 and 11,113 with EQ-5D-3L index values of 0.85 and 0.414, respectively, lower index would be chosen to construct AHC_worst_. In previous studies, the difference between the AHC_aggregate_ and AHC_worst_ was substantial in ages above 60 years suggesting that these AHCs deviate from the true acceptability threshold [7, 8].

To determine the location of the acceptability threshold, we adapted the EQ VAS instrument. In health valuation studies, multiple health states were recorded on a single EQ VAS [20]; therefore, we asked respondents to indicate the health state that is still acceptable in different ages on the same EQ VAS. To avoid suggesting a ranking on the vertical EQ VAS, we placed ages on a horizontal line at the midpoint of the EQ VAS and asked respondents to link each age and the VAS with a line. The adapted EQ VAS is shown in the Online Resource (Supplementary Fig. S2). We constructed AHCs from the acceptable EQ VAS scores at each age_AHS_, for which we use the term “VAS acceptable health curve (AHC_vas_)”.

At the end of the evaluation task, we asked respondents about whom they imagined when evaluating the acceptability of health states. Based on the reference person imagined, respondents were assigned to three categories: those who thought about themselves (“reference: oneself”), those who answered with reference to others (“reference: others”), and those who had both themselves and others in their mind (“reference: mixed”). The joint evaluation of the acceptability of EQ-5D-3L profiles using the adaptive testing algorithm will be described elsewhere.

### Statistical analysis

We summarised the key sample characteristics using descriptive methods. Then, we estimated the AHC_vas_ age profiles using a multilevel regression model. In the baseline model (Model 1, Eq. 1), AHC_vas_ was described by an intercept and a slope parameter, which were allowed to vary across individuals according the following equation:

1 $${\text{AHC}}_{\text{vasik }} = \alpha + \beta {\text{age}}_{\text{AHSik}} + \mu_{\text{i}} + \tau_{\text{i}} \; \times \;{\text{age}}_{\text{AHSik}} + \varepsilon_{\text{ik}} ,$$

where *age*_*AHSik*_ denotes age when acceptable health is evaluated by respondent *i* for the *k*th age. We centred age_AHS_ on 30 years; so, the intercept denoted by represents the population average level of acceptable health at age 30 (AHC_vas30_) and *μ* represents the individual-specific shifts in AHC_vas30_. The slope denoted by *β* represents the average acceptable deterioration rate of health with age (ADR) and *τ* stands for the individual-specific component of ADR. In the model, *μ* and *τ* were not estimated directly, but they were modelled as random effects, with only the variance of these parameters estimated.

To evaluate the effect of individual characteristics on acceptable health, the model was augmented by level-2 variables as follows:

2 $${\text{AHC}}_{\text{vasik}} = \alpha + \beta {\text{age}}_{\text{AHSik}} + \gamma X_{\text{i}} + \delta {\text{age}}_{\text{AHSik}} \; \times \;X_{\text{i}} + \mu_{\text{i}} + \tau_{\text{i}} \; \times \;{\text{age}}_{\text{AHSik}} + \varepsilon_{\text{ik}} ,$$

where *X*_i_ is the vector of individual characteristics of respondent *i*, the vectors of coefficients are *γ* and *δ* representing the effects of *X*_i,_ respectively, on the intercept and the slope. While holding *X* constant at the reference values, *α* and *β* represent the intercept and slope parameters, respectively. The individual-specific intercept and slope components that are not explained by *X* are represented by *μ* and *τ*, respectively.

We explored the effect of four sets of individual characteristics on the AHC_vas_. The model contained basic demographic characteristics, such as gender, age and education (Model 2). Respondents’ EQ VAS score and health-related lifestyle variables were included as a proxy for “experience with own health” (Model 3). We also included dummy variables to indicate respondents’ reference person when evaluating AHSs (Model 4). Finally, we explored the effect of the lifespan of close relatives and caregiver status as indicators for the “experience with others health” (Model 5).

In addition to the linear multilevel model, we also tested alternative AHC_vas_ models. We evaluated model fit by comparing residual variance ($\sigma_{\varepsilon }^{2}$) and Akaike’s information criterion (AIC). First, we compared Model 1 to a restricted specification including only a random intercept, but no random slope term, and a simple linear regression model containing no random terms. Model fit was substantially inferior for these restricted specifications ($\sigma_{\varepsilon }^{2}$ = 58.2, AIC = 8438 with random intercept only and $\sigma_{\varepsilon }^{2}$ = 199.7, AIC = 9320 with no random term, while $\sigma_{\varepsilon }^{2}$ = 17.2, AIC = 7657 for Model 1).

Second, we also compared Model 1 to a more general specification, allowing for nonlinear effects of age_AHS_:

3 $${\text{AHC}}_{\text{vasik}} = \alpha + \beta {\text{age}}_{\text{AHSik}} + \theta {\text{age}}_{\text{AHSik}}^{2} + \mu_{\text{i}} + \tau_{\text{i}} \; \times \;{\text{age}}_{\text{AHSik}} + \vartheta_{\text{i}} \; \times \;{\text{age}}_{\text{AHSik}}^{2} + \varepsilon_{\text{ik}} .$$

Although the likelihood ratio test was significant ($\chi_{{\left( {{\text{df}}\; = \;4} \right)}}^{2} = 187.1, \;p\; < \;0.0001)$ indicating better fit for the quadratic model vs the linear one (Model 1), the difference between predicted values were negligible, and the gain in fit was modest ($\sigma_{\varepsilon }^{2}$ = 10.9, AIC = 7478). Therefore, we favoured the linear model for more straightforward interpretation of the parameters.

## Results

### Sample characteristics

**Fig. 1:**
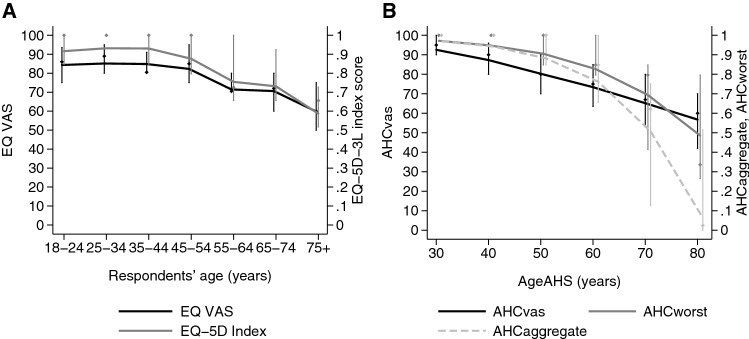
Respondents’ own health status and acceptable health curves. **a** own health measured by the EQ-5D-3L and EQ VAS; **b** acceptable health curves: AHC_vas_ (VAS acceptable health curve), AHC_aggregate_ (aggregate acceptable health curve), AHC_worst_ (worst acceptable health curve). Line graphs, diamonds and vertical lines indicate mean values, medians and interquartile ranges (IQR), respectively. The 25th percentile of AHC_aggregate_ at age_AHS_ 80 years was − 0.358 (truncated at 0 on the graph)

Altogether, 200 respondents participated in the study; however, only the 194 respondents were included in the analyses for whom any AHC_vas_ scores were available. Table 1 summarises the sample descriptive statistics. Mean age was 43.3 years (SD = 17.3), the youngest respondent was 19, and the eldest was 93 years old. Compared to the general population of Hungary, younger respondents, women, individuals with high education were slightly over-represented [21], over- and underweight status and high-risk alcohol intake were similar, while smoking and lack of exercise were somewhat less prevalent in our sample [22]. Respondents’ own health measured by the EQ-5D-3L index and EQ VAS scores are displayed in Fig. 1a. Full AHC_vas_ across all six age_AHS_ were available for 188 respondents (94%).

**Table 1:** Descriptive statistics of demographic characteristics and reference person when evaluating acceptable health

Variable	Category	*N*	%
Age	18–34	74	39.15
	35–64	90	47.62
	65+	25	13.23
Gender	Male	79	41.80
	Female	110	58.20
Education	Low/middle	77	40.96
	High	111	59.04
BMI	< 25	98	51.85
	≥ 25	91	48.15
Smoking	Yes	41	21.96
	No	148	78.31
High-risk alcohol	Yes	20	10.58
	No	169	89.42
Lack of exercise	Yes	105	55.56
	No	84	44.44
Relatives’ lifespan	< 75 years	70	35.18
	≥ 75 years	129	64.82
Informal caregiver	Yes	58	29.15
	No	141	70.85
Reference^a^	Oneself	88	47.31
	Others	59	31.72
	Mixed	39	20.97

^a^The person the respondent had in mind when considered the acceptability of health states for different ages

### Acceptability of health states

**Fig. 2:**
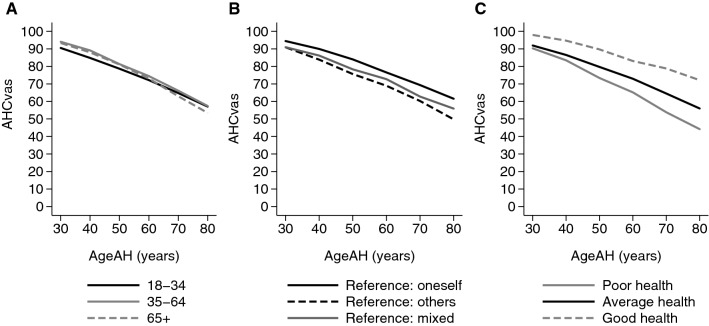
Acceptable health curves measured on EQ VAS (AHC_VAS_) by respondents’ subgroups. **a** By respondents’ age group; **b** by respondents’ reference person during the evaluation task; **c** by respondents’ own health; “reference: oneself“: respondents thinking of themselves during the evaluation task; “reference: other”: respondents thinking of others during the evaluation task; “reference: mixed”: respondents thinking of themselves and others during the evaluation task; “poor health”: ≤ sample mean EQ VAS − 1SD; “average health”: sample mean EQ VAS ± 1 SD; “good health”: ≥ sample mean EQ VAS + 1 SD

Acceptable health curves measured by EQ VAS score (AHC_vas_) and EQ-5D-3L index (AHC_aggregate_, AHC_worst_) are shown in Fig. 1b. For ages of 30, 40, 50, 60, 70 and 80 years, mean AHC_vas_ scores were 93, 87, 80, 73, 65 and 57, respectively. Both AHC_vas_ and the EQ-5D-3L index-based measures, AHC_aggregate_ and AHC_worst_ indicated that respondents considered health problems increasingly acceptable in older ages. However, the age profiles were markedly different. While AHC_aggregate_ indicated a rapid and nonlinear decline of acceptable health, AHC_worst_ and AHC_vas_ showed a modest nonlinear decline in similar range to respondents’ own EQ-5D-3L index scores, and own EQ VAS scores, respectively. We also observed differences between the EQ VAS-based and EQ-5D-3L index-based curves in their dispersion characteristics (Fig. 2a and b). The mean interquartile range (IQR) of EQ VAS by age group was 17 points (range 10–25); the mean IQR of AHC_vas_ by age_AHS_ group was rather similar: 19 points (range 10–28). However, the average IQR by age or age_AHS_ group was 0.19 (range 0.152–0.344), 0.22 (range 0–0.532), 0.33 (range 0–0.874) for EQ-5D-3L index, AHC_worst_ and AHC_aggregate_, respectively. The dispersion for all measures was greatest in older age_AHS_ groups.

Next, we explored the differences of AHC_vas_ between subgroups of the sample. Figure 2a shows the AHC_vas_ by respondents’ age groups. The AHC_vas_ profiles were similar for the three age groups. Both the youngest and oldest respondents considered somewhat lower levels of health acceptable for their own age_AHS_ groups. Figure 2b compares AHC_vas_ for the three types of respondents based on the reference person they imagined during the evaluation task. Those who imagined themselves when evaluating AHSs (“reference: oneself” group) considered the least health problems acceptable. Moreover, AHC_vas_ declined more rapidly in the “reference: others” group compared to the “reference: oneself” or “reference: mixed” groups.

We also explored how respondents’ health affected their AHC_vas_ (Fig. 2c). The data suggest that respondents who were healthier compared to the sample average (higher EQ VAS scores), accepted less health problems (higher AHC_vas_)  than the ones who indicated more subjective health problems on the EQ VAS.

### Factors affecting the acceptability of health states

Table 2 presents the results of multilevel regression estimates. The baseline model (M1) confirmed that there were significant differences in both the level of AHC_vas_ and the slope of age_AHS_ among respondents. The estimated level of AHC_vas_ at age_AHS_ 30 years had a mean of 93.4, with a significant variance over respondents indicated by the random effect (SD 8.7 points, *p* < 0.001). AHC_vas_ decreased by 7.2 points with a 10-year increase in age_AHS_ on average, while the SD of ADR per 10 years was 3.4 points (*p* < 0.001). When interpreting the regression results, lower intercept (lower AHC_vas_ at age_AHS_ 30) and smaller slope coefficients (greater ADR) indicate more acceptable health problems.

**Table 2:** Multilevel regression models of AHCvas

	M1	M2	M3	M4	M5
Level-1 parameters					
Constant	93.87*** (0.662)	95.76*** (1.481)	92.71*** (1.770)	94.39*** (1.856)	96.71*** (2.037)
Age_AHS_	− 0.723*** (0.026)	− 0.757*** (0.059)	− 0.751*** (0.071)	− 0.696*** (0.075)	− 0.734*** (0.082)
Level-2 parameters: intercept					
Age: 18–34		− 4.143*** (1.398)	− 4.654*** (1.315)	− 4.564*** (1.312)	− 4.636*** (1.318)
Age: 65+		− 0.148 (2.023)	3.372* (1.968)	2.472 (1.969)	2.673 (2.000)
Female		0.687 (1.317)	0.864 (1.278)	0.660 (1.269)	0.686 (1.257)
Education: tertiary		− 1.053 (1.321)	− 1.238 (1.212)	− 1.026 (1.204)	− 0.856 (1.193)
Current health^a^			0.280*** (0.047)	0.268*** (0.046)	0.271*** (0.046)
High-risk alcohol			1.032 (2.056)	0.406 (2.038)	0.0466 (2.012)
Smoking			2.114 (1.557)	2.320 (1.538)	2.553* (1.527)
Lack of exercise			1.024 (1.244)	0.796 (1.238)	0.305 (1.243)
BMI 25+			3.220** (1.312)	3.455*** (1.295)	3.501*** (1.289)
Reference: mixed				− 2.844* (1.577)	− 2.825* (1.585)
Reference: others				− 3.101** (1.377)	− 2.640* (1.368)
Relatives’ lifespan 75+					− 3.061** (1.272)
Informal caregiver					− 1.123 (1.415)
Level-2 parameters: slope					
Age_AHS_ × age: 18–34		0.083 (0.056)	0.066 (0.053)	0.075 (0.053)	0.104* (0.053)
Age_AHS_ × age: 65+		− 0.055 (0.080)	0.058 (0.079)	0.036 (0.080)	− 0.018 (0.081)
Age_AHS_ × female		− 0.011 (0.052)	0.009 (0.052)	0.012 (0.052)	0.002 (0.051)
Age_AHS_ × education: tertiary		0.023 (0.052)	0.007 (0.049)	0.012 (0.049)	0.012 (0.048)
Age_AHS_ × current health^a^			0.007*** (0.002)	0.007*** (0.002)	0.007*** (0.002)
Age_AHS_ × high-risk alcohol			0.150* (0.083)	0.132 (0.083)	0.143* (0.081)
Age_AHS_ × smoking			− 0.022 (0.063)	− 0.023 (0.063)	− 0.042 (0.062)
Age_AHS_ × lack of exercise			− 0.125** (0.050)	− 0.132*** (0.050)	− 0.140*** (0.050)
Age_AHS_ × BMI 25+			0.089* (0.053)	0.090* (0.053)	0.070 (0.052)
Age_AHS_ × reference: mixed				− 0.056 (0.064)	− 0.034 (0.065)
Age_AHS_ × reference: others				− 0.138** (0.056)	− 0.142** (0.055)
Age_AHS_ × relative’s lifespan 75+					0.007 (0.052)
Age_AHS_ × informal caregiver					0.166*** (0.057)
Random effect parameters					
Variance (age_AHS_)	0.117*** (0.013)	0.116*** (0.013)	0.097*** (0.011)	0.095*** (0.011)	0.091*** (0.011)
Variance (constant)	75.483*** (8.697)	70.870*** (8.257)	56.961*** (6.813)	54.746*** (6.633)	52.875*** (6.446)
Covariance (constant, age_AHS_)	0.041 (0.238)	0.149 (0.230)	− 0.234 (0.197)	− 0.320* (0.194)	− 0.283 (0.188)
AIC	7657.354	7624.635	7522.899	7401.364	7352.859
Observations	1145	1139	1133	1115	1109
Number of groups	194	193	192	189	188

Standard errors in parentheses

***p < 0.01, **p < 0.05, *p < 0.1

^a^Current health is measured on the EQVAS scale, centred on the sample mean

We estimated the effect of individual characteristics on AHC_vas_ by adding four groups of explanatory variables to the base model in a stepwise manner. Altogether, individual characteristics accounted for a moderate share of the variation in the slope and level of AHC_vas_. Compared with the baseline model, in the full specification (M5), 2/3 of the variance of the intercept and ¾ of the variance of the slope parameter remained unexplained.

Compared to the reference group (35–64), young respondents reported a lower level of acceptable health. Education and gender had no detectable effect. ADR was only minimally affected by demographic variables. Current health status, measured on the EQ VAS, was associated with both the level and the slope of the age profiles. Healthier respondents tended to accept a less rapid deterioration of health with age, and also considered a higher level of health acceptable at the age_AHS_ of 30. A one SD difference in current health implied 4 points higher AHC_vas_ at the age_AHS_ of 30, and 1.1 points smaller ADR per 10 years. Lifestyle variables had a weak effect. Lack of exercise and high BMI were associated with, respectively, greater ADR and higher level of AHC_vas_, in line with the expectation of more health problems.

**Fig. 3:**
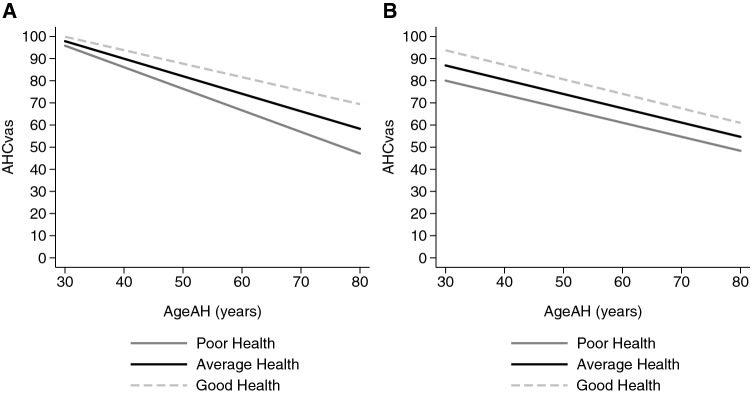
Predicted AHC_vas_ for reference person subsamples by respondents’ own health. **A** “Reference: oneself”: respondents thinking of themselves during the evaluation task; **b** “reference: other and mixed”: respondents thinking of others, or others and themselves; predicted AHC_vas_ for male, age 35–64, education: middle or low, with no health-related risks; “poor health”: ≤ sample mean EQ VAS − 1 SD; “average health”: sample mean EQ VAS ± 1 SD; “good health”: ≥ sample mean EQ VAS + 1 SD

In Model 4, the reference person when evaluating acceptability was also added. The results confirm the pattern of Fig. 3b. Respondents who evaluated health states with respect to themselves set the level of AHC_vas_ higher at the age_AHS_ of 30 and were willing to accept only a less rapid decline with age than respondents evaluating acceptability with reference to others. Those with a mixed reference were in between these two types.

Finally, relative’s longer lifespan was associated with a lower level of AHC_vas_, while respondents with a caregiver experience reported a smaller ADR.

Next, we explored the effect of the reference person when evaluating acceptability, current health and age in more detail. We re-estimated the models M2 and M3 for two subgroups: respondents, who thought only about themselves (reference: oneself) and the ones who also thought about others (reference: others or reference:mixed groups).  (Table 3) Current health had a smaller effect on the level of AHC_vas_ at the age_AHS_ of 30 in case of respondents thinking of themselves than in the other or mixed reference subsample. At the same time, better current health was associated with greater ADR in the first subsample only. These differences are shown by Fig. 3. Predicted AHC_vas_ values were calculated for three otherwise identical representative individuals with poor, average and good current health, for both subsamples. Respondents in poor health and thinking of themselves accepted a stronger decline of health with age.

**Table 3:** Multilevel regression models of AHC_VAS_ for subsamples by reference person when evaluating acceptable health problems

	Reference: oneself		Reference: others or mixed	
	M2	M3	M2	M3
Level-1 parameters				
Constant	98.92*** (1.741)	97.87*** (2.294)	92.18*** (2.496)	86.91*** (2.580)
Age_AHS_	− 0.732*** (0.081)	− 0.792*** (0.095)	− 0.759*** (0.086)	− 0.645*** (0.102)
Level-2 parameters: intercept				
Age: 18–34	− 4.255** (1.918)	− 4.593** (1.934)	− 3.526* (2.013)	− 3.344** (1.698)
Age: 65+	− 2.401 (2.288)	− 0.168 (2.337)	1.712 (3.673)	7.355** (3.491)
Current health^i^		0.137** (0.058)		0.463*** (0.070)
Level-2 parameters: slope				
Age_AHS _× age: 18–34	0.183** (0.089)	0.121 (0.080)	0.010 (0.070)	− 0.006 (0.068)
Age_AHS _× age: 65+	− 0.022 (0.106)	0.170* (0.096)	− 0.216* (0.126)	− 0.277** (0.138)
Age_AHS _× current health^i^		0.012*** (0.002)		− 0.001 (0.003)
Observations	532	532	589	583
Number of groups	89	89	101	100

Standard errors in parentheses

Specifications identical to M2 and M3 of Table 2

***p < 0.01, **p < 0.05, *p < 0.1

^a^Current health is measured on the EQVAS scale, centred on the sample mean

## Discussion

To our knowledge, this is the first study that explored acceptable health states using the EQ VAS instrument. We found that the modified EQ VAS was a feasible and convenient measure of AHSs for vast majority of our respondents. Our findings confirm previous research results that people find worsening of health with age acceptable [7–9]. The core hypothesis is that respondents have internal age-dependent reference points against which they judge the acceptability of health states.

Previous studies measured AHSs using the descriptive system of the EQ-5D-3L instrument. In these studies, AHSs were assessed separately by the five dimensions of the EQ-5D-3L, and assumptions were made about the acceptability simultaneous health problems in more than one EQ-5D-3L dimension. Although the acceptability of a few EQ-5D-3L health states profiles was also evaluated, current knowledge is limited about how people jointly evaluate the acceptability of simultaneous problems in multiple dimensions. One of the benefits of using the EQ VAS is that it provides a single score about where acceptable health states lie compared to the best and worst imaginable overall health status. The moderate slope of the AHC_vas_ with age suggested that by evaluating the acceptability of health states globally, in older ages people are less likely to accept as many combined problems as suggested by the AHC_aggregate_. Moreover, the dispersion of AHC_vas_ was well below the variation of EQ-5D-3L descriptive system-based AHCs, suggesting that using EQ VAS may allow for defining and detecting acceptability thresholds more precisely. While the dispersion of AHC_vas_ was similar to that of the measures of current health, the greater dispersion of AHC_aggregate_ suggests that considerable measurement error arises from the construction of AHCs from the separately evaluated AHSs by dimension. However, since the AHC_aggregate_ is artificially constructed from EQ-5D-3L index values, which reflect societal preferences rather than each respondents’ global evaluation of individual health [20], the AHC_aggregate_ and AHC_vas_ are not directly comparable measures of AHSs.

Although it may be hypothesised that underlying health preferences influence the acceptability of health problems, the evaluation exercise of acceptable health problems is different in many aspects from health-state valuation tasks: it does not involve assumptions about death, expected lifespan, nor trade-off or risk-evaluation is involved [12, 23]. Although the cognitive processes behind the evaluation exercise of acceptable health are yet to be explored, our results confirm previous findings that respondents’ age, health status, lifestyle, and other health-related experiences, such as informal caregiver status or the lifespan of close relatives influence the internal reference for acceptable health.

We found that ceteris paribus, the younger respondents reported in younger ages more, and in older ages less health problems acceptable compared to older ones. Other studies demonstrated greater acceptability of health problems by elderly respondents in the general population [7], and in patients with RA [9]. In our study, worse current health of respondents was associated with more acceptable health problems. In the Dutch general population study, suffering from a severe disorder or having a chronic condition was not associated with the acceptability of health problems [7], while lower EQ VAS scores of Hungarian RA patients predicted more acceptable health problems [9]. Patients’ adaptation to their conditions affects the valuation of health states [24] which may also increase the acceptability of non-perfect health by older individuals experiencing health problems.

In addition to older respondents, the younger age group also deserves attention. The relationship of age and health-state utilities was bell-shaped in the UK TTO EQ-5D valuation study [25] and we found increased acceptability of health problems in the 18–34-year-old age group. Perceived severity of health outcomes may play a role in the prevention of health-risk behaviours [26], an important health concern among young adults [27]. Acceptability of health problems may be linked to the perception of health risks by younger adults, although this hypothesis needs empirical testing. Therefore, the complex interplay between the effects of respondents’ health and age on the acceptability of health problems requires further investigation, which may provide useful insights to health valuation as well as health prevention research.

Lifestyle-related variables influenced the acceptability of health problems in both our sample and the Dutch general population, albeit in different ways. In the Dutch population, healthy diet was associated with significantly fewer acceptable health problems. In our sample, lack of exercise was associated with more (greater ADR), while high BMI and high-risk alcohol intake were associated with less acceptable health problems. Longer lifespan of close relatives was associated with more acceptable health problems in our study, while had opposite effect in the Dutch study. We also found that being a caregiver was associated with less AHSs (via lower ADR). These findings warrant deeper qualitative investigation of the experiences and attitudes about own and others’ health and the context that shape the acceptability of health problems.

We find important to note that the effects of explanatory variables need to be interpreted in the light of the potentially different measurement properties of our methods as well as different preferences of the studied populations. Wouters et al. recruited an online sample of the Dutch general population (*n* = 1067), and constructed an area under curve (AUC) AHC_aggregate_ values between 40 years of age_AHS_ and the expected lifespan of respondents to study the effect of explanatory variables on AHSs [7]. We conducted computer-assisted interviews in the Hungarian population, and evaluated the level and slope parameters of the AHC_vas_ separately as measures of AHSs.

In addition to the four scenarios of health preference evaluation (general public vs patients, own vs hypothetical health states), the evaluation of AHSs in different ages involves a new hypothetical situation [23]. Our study provided insights about people’s opinion formulating mechanisms when evaluating AHSs. Despite the neutral question, approximately half of the respondents imagined themselves, one-third others and one-fifth both themselves and others during the evaluation task. Mulhern et al. [28] found similar respondent subtypes when evaluating hypothetical health states. We also found that the reference person respondents imagined affected the way respondent’s age and health status influenced the evaluation of AHSs, which warrants further investigation as well as methodological refinement of the evaluation exercise.

Our research has three important limitations. First, our sample was a small non-representative sample of the Hungarian population, which limits the generalisation of our findings. Second, although the EQ VAS has been validated for measuring the current health status of responders, its psychometric properties has not been formally tested and validated for the measurement of AHSs. For instance, respondents may have provided systematic answers on the single VAS scale, which needs further exploration. Also, because some authors raised theoretical concerns about using the EQ VAS for health economic evaluation [12] the theoretical and ethical basis how VAS-based measures of AHSs could inform decision-making have yet to be determined. However, our results showed that EQ VAS has promising properties in the evaluation of AHSs, warranting further exploration of the acceptable health concept.

## Conclusions

We measured AHSs in different ages by adapting the EQ VAS, which proved to be a convenient and feasible measure. Our findings confirmed that health problems are increasingly acceptable with age. The comparison of VAS-based and EQ-5D-3L index-based measures suggested that the amount of acceptable health deterioration with age depends on the measurement method. In our sample, respondents’ age, health status, lifestyle, and other health-related experiences influenced the evaluation of acceptable health problems. We also found that people imagined themselves, others or both during the evaluation of acceptable health, which also influenced the evaluation of AHSs. However, our non-representative sample limits the generalizability of our findings.

AHSs may indicate societal preferences about the severity of disease and age in a single measure, and may serve as a reference point in healthcare priority setting. However, measuring AHSs need further theoretical and methodological exploration, before their practical application can be considered.

## Electronic supplementary material

Below is the link to the electronic supplementary material.

Supplementary material 1 (PDF 89 kb)

Supplementary material 2 (PDF 105 kb)
