# Supplementary material for: Determinants of the acceptability of health problems in different ages: exploring a new application of the EQ VAS
Source: Eur J Health Econ. 2019 May 20;20(Suppl 1):31–41. doi: 10.1007/s10198-019-01060-3 (PMC6544591; doi:10.1007/s10198-019-01060-3)
Supplement: Supplementary file 1 — Supplementary material 1 (PDF 89 kb) [file 10198_2019_1060_MOESM1_ESM.pdf]

Determinants of the acceptability of health problems in different ages: exploring a new application of the EQ VAS

Authors
Zsombor Zrubka, Zoltán Hermann, László Gulácsi, Valentin Brodszky, Fanni Rencz, Márta Péntek

Correspondence: Zsombor Zrubka, Department of Health Economics, Corvinus University of Budapest, Fővám tér 8., H-1093 Budapest, Hungary e-mail: zsombor.zrubka@un-corvinus.hu, tel: +36-1-482-5308

Journal: European Journal of Health Economics

Supplementary Figure S1. Sample question of acceptable health problems by EQ-5D-3L dimensions

- *Can you indicate beyond what age you consider the specified level of problems with 'mobility' to be acceptable?* (Please indicate below the relevant age categories)

	Age category						
	Beyond the age of 30	Beyond the age of 40	Beyond the age of 50	Beyond the age of 60	Beyond the age of 70	Beyond the age of 80	Never
Some problems with walking							
Confined to bed							

Ref: Brouwer et al. (2005) [8], replicated with copyright from the publisher Elsevier, under license number 4547720239361
