# Supplementary material for: Determinants of the acceptability of health problems in different ages: exploring a new application of the EQ VAS
Source: Eur J Health Econ. 2019 May 20;20(Suppl 1):31–41. doi: 10.1007/s10198-019-01060-3 (PMC6544591; doi:10.1007/s10198-019-01060-3)

Determinants of the acceptability of health problems in different ages: exploring a new application of the EQ VAS

Authors

Zsombor Zrubka, Zoltán Hermann, László Gulácsi, Valentin Brodszky, Fanni Rencz, Márta Péntek

Correspondence: Zsombor Zrubka, Department of Health Economics, Corvinus University of Budapest, Fővám tér 8., H-1093 Budapest, Hungary e-mail: zsombor.zrubka@un-corvinus.hu, tel: +36-1-482-5308

Journal: European Journal of Health Economics

Supplementary Figure S2. Modified EQ VAS to measure acceptable health states in different ages

To help people say how good or bad their state of health is, we have drawn a scale (rather like a thermomether) on which the best state you can imagine is marked 100 and the worst state you can imagine is marked 0.

We would like you to indicate on this scale how good or bad in your opinion is the **health state that is still acceptable at 30, 40, 50, 60, 70 and 80 years of age.** Please do this by drawing a line from the ages next to the box (on which the statement is written: „still acceptable health state") to whichever point on the scale indicates how good or bad the still acceptable health state is in that age.

Still acceptable health state

30 – 40 – 50 – 60 – 70 - 80

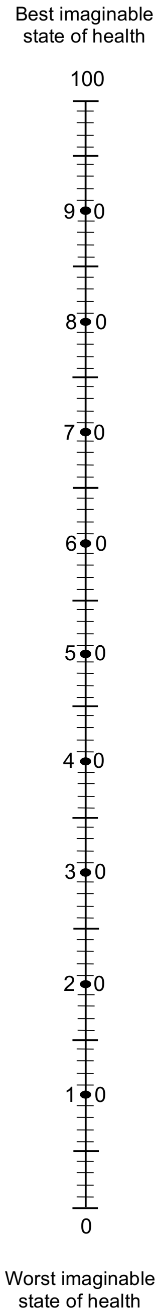

Supplement: Supplementary file 2 — Supplementary material 2 (PDF 105 kb) [file 10198_2019_1060_MOESM2_ESM.pdf]
